# LOOK WHO’S TALKING: TRADITIONAL AND ELECTRONIC MEDIUMS OF CONTACT LINKED WITH LATER-LIFE SIBLING RELATIONSHIPS

**DOI:** 10.1093/geroni/igz038.1191

**Published:** 2019-11-08

**Authors:** Melanie S Hill, Jeremy B Yorgason, Sarah Coyne, Alexander C Jensen

**Affiliations:** Brigham Young University, Provo, Utah, United States

## Abstract

The sibling role is often the longest lasting relationship between individuals. As such, older adults may turn to siblings in later life as it is a relationship that is already familiar. Having a close and less conflictual relationship with a sibling may be especially important as older adults value siblings for emotional and practical support exhibited through contact. Minimal research has examined mediums of contact used between sibling dyads despite the increase use in technology among older adults. Using a sample of 491 Americans (Mage = 58.96) recruited via Amazon Mechanical Turk (Mturk), the current study examined five mediums of contact (i.e., in person, telephone, e-mail, texting, and social media) and how each type independently is related to sibling closeness and conflict. Further, using regression analyses in STATA, two and three-way interactions were examined to assess the role of sibling dyad composition affecting this relationship. Results indicated that contact through telephone was associated with higher sibling closeness for all sibling dyads, and that association was stronger for females with a sister compared to males with a brother. Further, in person and texting contact was especially beneficial for females with a brother. Main effects revealed contact in person, via social media, over the telephone, or through email, reported more sibling closeness, while those who engaged in more email contact reported less conflict. Thus, even in later life, siblings are keeping in contact with one another through both traditional and electronic mediums of communication, and this contact appears especially beneficial for sisters.

